# Public Health Surveillance Studies of Alcohol Industry Market and Political Strategies: A Systematic Review

**DOI:** 10.15288/jsad.2019.80.149

**Published:** 2019-04-24

**Authors:** Jim McCambridge, Rachel Coleman, Julie McEachern

**Affiliations:** ^a^ Department of Health Sciences, University of York, Heslington, York, United Kingdom

## Abstract

**Objective:**

This review examines public health surveillance (PHS) studies of alcohol industry actors that explore the implications of the integration of business and political strategies for public health.

**Method:**

Eligible for inclusion were studies published in English language peer-reviewed journals since 1980 that sought to investigate both alcohol industry business and political strategies and their implications for public health. Studies were also required to present economic, political, and health data together. Seven databases were searched until May 2018.

**Results:**

Six studies were identified as eligible for inclusion in this review, undertaken in high-, middle-, and low-income countries and published between 2000 and 2015. Political strategies are driven largely by business interests, whether at the company, sectoral, or industry level, and corporate social responsibility activities may be integrated within overall strategies. There is a high degree of collaboration in political strategy development between companies, facilitated by growing concentration among global producers operating in increasingly oligopolistic markets. There are limited insights into the dynamics of market competition and limited methodological data available.

**Conclusions:**

PHS studies play a valuable role in identifying aspects of alcohol industry strategies that warrant more detailed and carefully designed research, as well as in elucidating global health implications. Further research in PHS and other kinds of studies will assist efforts to reduce the global burden of disease caused by alcohol.

The alcohol policies most likely to be effective in reversing the growing burden of noncommunicable diseases (NCDs) and the adverse impacts of alcohol on society are those that reduce affordability and availability and the cultural acceptability of heavy drinking and intoxication (Babor, 2010; World Health Organization [WHO], 2010). The activities of industry actors have been identified as key reasons for the lack of implementation of effective policy measures (Jernigan & Trangenstein, 2017). Adoption and implementation of effective policies has been very limited in low- and middle-income countries (LMICs) where major commitments are needed if the Sustainable Development Goals with respect to the harmful use of alcohol are to be met (Collin & Casswell, 2016; Jernigan & Trangenstein, 2017).

The WHO defines public health surveillance (PHS) as “the continuous, systematic collection, analysis and interpretation of health-related data needed for the planning, implementation, and evaluation of public health practice” (WHO, n.d.). Babor and colleagues have drawn attention to the need for this approach to be applied to monitoring alcohol industry actors because of their position as key inducers of NCDs (Babor, 2016; Babor & Robaina, 2013; Jahiel & Babor, 2007). When transnational corporations identify particular LMICs or regions for sales growth, it is expected that both overall consumption and harmful drinking will rise. For example, sub-Saharan Africa is attractive to alcohol companies, as almost half the population is under 14 years old and only about 30% of adults drink alcohol (Ferreira-Borges et al., 2017). This has led to major mergers within the industry (Collin et al., 2015), and rising alcohol consumption. The adverse consequences, entirely predictable and avoidable, are epidemics of death and disease and the attendant social costs (Ferreira-Borges et al., 2015).

It is understood that transnational corporations generally integrate their market and political strategies (Hillman, 2003; Hillman & Hitt, 1999), seeking to influence policy to advance business interests, and this would appear to have been the case in Africa with the alcohol industry (Ferreira-Borges et al., 2015, 2017). Corporate social responsibility (CSR) initiatives may also be integrated with other business and political components in corporate strategies (Jones et al., 2016; Mart & Giesbrecht, 2015). PHS studies thus could either examine specific components within the overall strategies of corporate actors or investigate their integration. Recent reviews are available that examine various aspects of alcohol company marketing (e.g., Noel et al., 2017), and systematic reviews of CSR (Mialon & McCambridge, 2018) and political strategies (McCambridge et al., 2018). However, no systematic review exists that examines studies of the integration of such elements in strategic decision-making by alcohol industry actors.

An epidemiological cascade model (Babor & Robaina, 2013) has been developed based on Jahiel’s corporation-induced disease theory (Jahiel, 2008; Jahiel & Babor, 2007) linking economic and political factors to alcohol harms. This model posits that business and political activities of alcohol industry actors are key underlying drivers of alcohol-related harms, and as such require intervention in order to reduce population-level harms. As the need for PHS studies that address the epidemiological cascade for alcohol has recently been explicitly identified, it was expected at the outset that this review would identify an emerging literature. The present study provides a basis for examining how far evidence in PHS studies bears upon integrated industry strategies, as well as the implications for future research.

## Method

This review seeks to examine PHS studies of alcohol industry actors as upstream determinants of population health. This study will pay particular attention to PHS studies that focus on the integration of business and political strategies and explore their implications for public health. The aims are to provide an overview of this literature and to develop hypotheses on the relationships between the business and political strategic operations of alcohol companies that have identified implications for population health.

Study objectives are (a) to examine the nature of the data sources and methods used in PHS studies of alcohol industry actor integrated strategies; (b) to identify the nature of the data available in such PHS studies on economic interests, involvements in policy making, CSR, and population health; (c) to examine the integration of market, CSR, and political strategies of individual companies and/or the industry as a whole, and thus the nature of alcohol industry strategies; (d) to identify pathways to, and impacts on, population health, particularly elucidation of the public health implications for LMICs; and (e) to consider the need for further research.

To be included in this review, studies must be published in peer-reviewed journals since 1980; published in the English language; conceptualized as PHS studies of industry actors that seek to investigate the upstream economic and political determinants of alcohol consumption, harms, and/or population health (e.g., as stated in study aims or introduction); empirical investigations presenting economic (e.g., industry structure or marketing or product design) and political (e.g., lobbying or policy goals) data on alcohol industry actors; providing public health data (e.g., alcohol consumption and/or harms), and/or have a major study focus on analyzing public health implications.

Excluded are studies that do not provide data specifically on alcohol industry actors (separately where other corporate sectors are also studied) and PHS or other studies not both seeking to study and presenting data on economic, political, and health issues together. Studies of either market or political strategies alone are thus not eligible for inclusion.

We selected 1980 to include data before the global concentration of alcohol producers since the 1990s (Jernigan, 2009). Literature search strategies have been developed using both Medical Subjects Headings (MeSH) terms and key words. Seven health, social science, and business databases were searched as follows:

- Web of Science Core Collection (Web of Science interface);
- CINAHL Plus (EBSCOhost interface);
- Business Source Premier (EBSCOhost interface);
- Embase (Ovid interface);
- MEDLINE (Ovid interface);
- PsycINFO (Ovid interface); and
- Scopus (Scopus interface).

Each database was searched from 1980 onward in two waves of searching. Databases were last searched on May 15, 2018. Searching also included backward and forward citation searches, using the references cited in included data sources and Web of Science, respectively. Nondatabase searches comprised hand searching of the journal *Addiction* and contacts with topic experts. That particular journal was chosen on the basis of knowledge of the topic area, and hand searching undertaken to inform assessment of the adequacy of the database searches.

The basic search strategy was organized around the three constructs of “alcohol,” “industry,” and “public health” and developed with the support of a specialist librarian. The search strategy for MEDLINE is presented in Table 1.

**Table 1.**
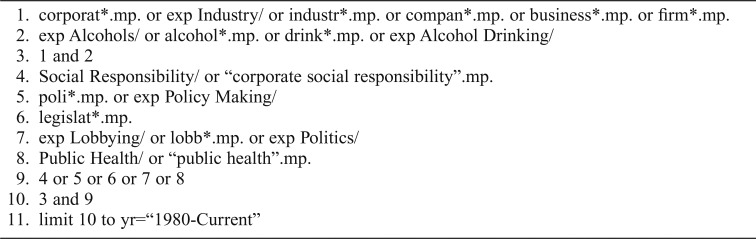
MEDLINE search strategy

1. corporat*.mp. or exp Industry/ or industr*.mp. or compan*.mp. or business*.mp. or firm*.mp.
2. exp Alcohols/ or alcohol*.mp. or drink*.mp. or exp Alcohol Drinking/
3. 1 and 2
4. Social Responsibility/ or “corporate social responsibility”.mp.
5. poli*.mp. or exp Policy Making/
6. legislat*.mp.
7. exp Lobbying/ or lobb*.mp. or exp Politics/
8. Public Health/ or “public health”.mp.
9. 4 or 5 or 6 or 7 or 8
10. 3 and 9
11. limit 10 to yr=“1980-Current”

The material retrieved was downloaded and imported into EndNote. Duplicates were removed using this software. Titles and abstracts were initially screened by one researcher. Potentially eligible full texts were obtained and eligibility determined separately by two researchers, with any disagreements resolved through discussions with a third researcher as necessary.

Data were analyzed by identifying material within studies that addressed individual study objectives, presented in order of the objectives. For Objective 3, data were thematically analyzed within and across studies using approaches appropriate for systematic review data (Thomas & Harden, 2008). Examination of issues pertaining to risk of bias is handled in the Discussion section. There is no published protocol for this review.

## Results

**Figure 1.**
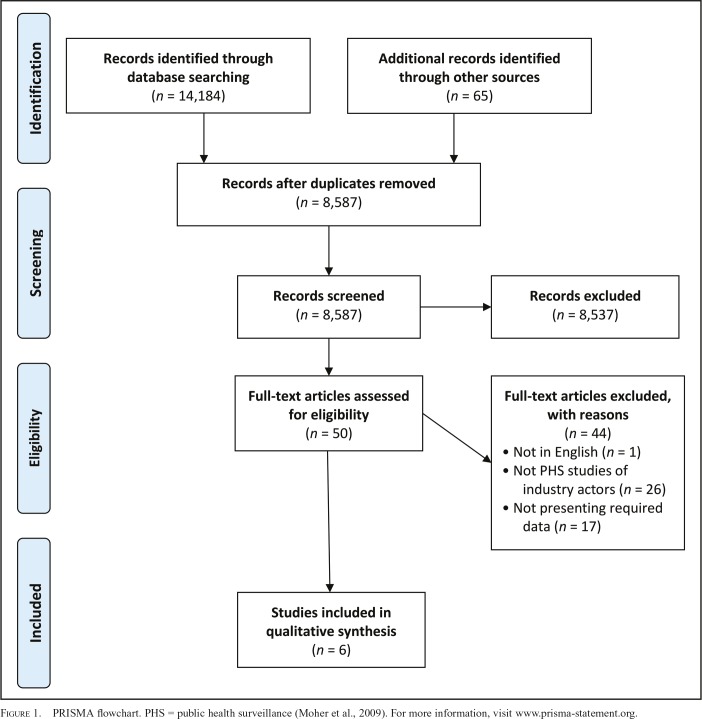
. PRISMA flowchart. PHS = public health surveillance (Moher et al., 2009). For more information, visit www.prisma-statement.org.

Six studies were identified as eligible for inclusion in this review (see the PRISMA flowchart in Figure 1). These study alcohol industry activity in Canada and the United States (Giesbrecht, 2000, 2006), Australia (Stockwell & Crosbie, 2001), India (Esser & Jernigan, 2015), and the continent of Africa (Babor et al., 2015; Jernigan & Babor, 2015), and only one investigates a company (Diageo; Esser & Jernigan, 2015). Three studies are somewhat older (Giesbrecht, 2000, 2006; Stockwell & Crosbie, 2001), and three are more recent (Babor et al., 2015; Esser & Jernigan, 2015; Jernigan & Babor, 2015).

### Methods and the nature of the data

Table 2 provides a summary of the characteristics of the included studies. No study identified itself as a PHS study, and there is scant information provided on study design or analysis. Some information was available on data sources and collection methods, although largely not to the extent of being reproducible. The limited methodological data available means that it is not possible to assess the strength of the study findings in light of the methods of data collection or analysis. Table 3 provides an overview of the data available in the included studies.

**Table 2.**
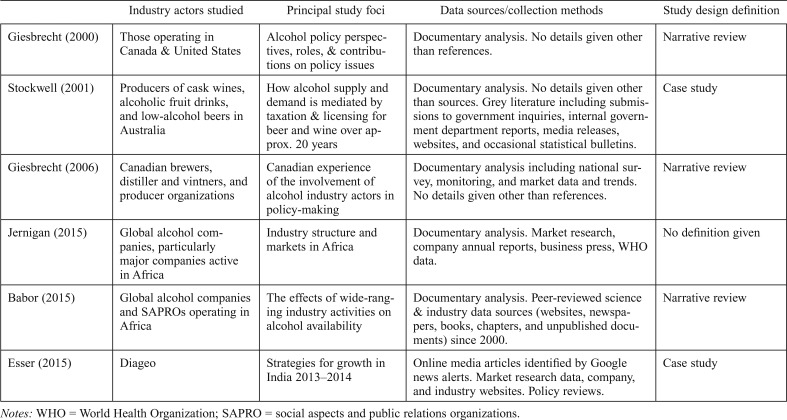
Characteristics of included studies

	Industry actors studied	Principal study foci	Data sources/collection methods	Study design definition
Giesbrecht (2000)	Those operating in Canada & United States	Alcohol policy perspectives, roles, & contributions on policy issues	Documentary analysis. No details given other than references.	Narrative review
Stockwell (2001)	Producers of cask wines, alcoholic fruit drinks, and low-alcohol beers in Australia	How alcohol supply and demand is mediated by taxation & licensing for beer and wine over approx. 20 years	Documentary analysis. No details given other than sources. Grey literature including submissions to government inquiries, internal government department reports, media releases, websites, and occasional statistical bulletins.	Case study
Giesbrecht (2006)	Canadian brewers, distiller and vintners, and producer organizations	Canadian experience of the involvement of alcohol industry actors in policy-making	Documentary analysis including national survey, monitoring, and market data and trends. No details given other than references.	Narrative review
Jernigan (2015)	Global alcohol companies, particularly major companies active in Africa	Industry structure and markets in Africa	Documentary analysis. Market research, company annual reports, business press, WHO data.	No definition given
Babor (2015)	Global alcohol companies and SAPROs operating in Africa	The effects of wide-ranging industry activities on alcohol availability	Documentary analysis. Peer-reviewed science & industry data sources (websites, newspapers, books, chapters, and unpublished documents) since 2000.	Narrative review
Esser (2015)	Diageo	Strategies for growth in India 2013–2014	Online media articles identified by Google news alerts. Market research data, company, and industry websites. Policy reviews.	Case study

*Notes:* WHO = World Health Organization; SAPRO = social aspects and public relations organizations.

**Table 3.**
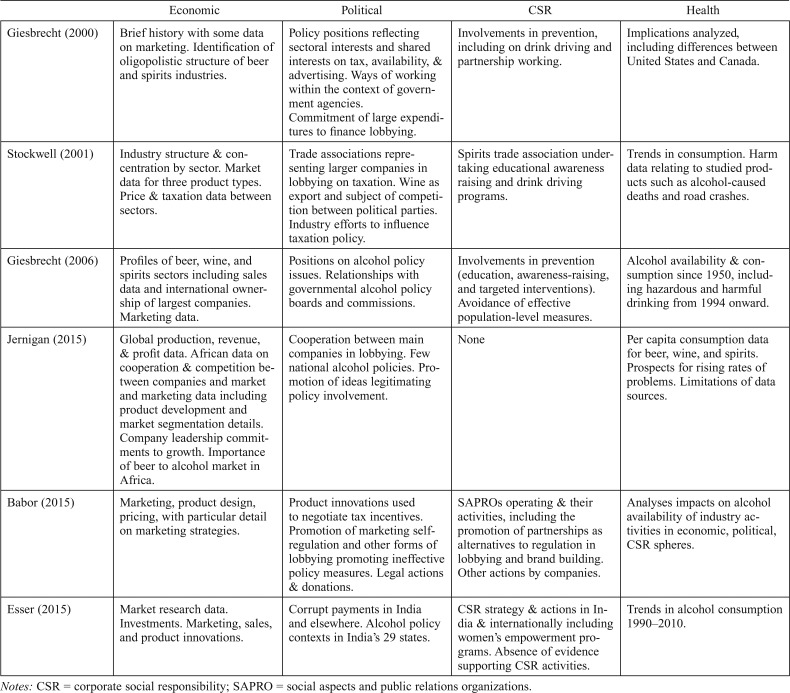
Overview of the nature of data presented

	Economic	Political	CSR	Health
Giesbrecht (2000)	Brief history with some data on marketing. Identification of oligopolistic structure of beer and spirits industries.	Policy positions reflecting sectoral interests and shared interests on tax, availability, & advertising. Ways of working within the context of government agencies. Commitment of large expenditures to finance lobbying.	Involvements in prevention, including on drink driving and partnership working.	Implications analyzed, including differences between United States and Canada.
Stockwell (2001)	Industry structure & concentration by sector. Market data for three product types. Price & taxation data between sectors.	Trade associations representing larger companies in lobbying on taxation. Wine as export and subject of competition between political parties. Industry efforts to influence taxation policy.	Spirits trade association undertaking educational awareness raising and drink driving programs.	Trends in consumption. Harm data relating to studied products such as alcohol-caused deaths and road crashes.
Giesbrecht (2006)	Profiles of beer, wine, and spirits sectors including sales data and international ownership of largest companies. Marketing data.	Positions on alcohol policy issues. Relationships with governmental alcohol policy boards and commissions.	Involvements in prevention (education, awareness-raising, and targeted interventions). Avoidance of effective population-level measures.	Alcohol availability & consumption since 1950, including hazardous and harmful drinking from 1994 onward.
Jernigan (2015)	Global production, revenue, & profit data. African data on cooperation & competition between companies and market and marketing data including product development and market segmentation details. Company leadership commitments to growth. Importance of beer to alcohol market in Africa.	Cooperation between main companies in lobbying. Few national alcohol policies. Promotion of ideas legitimating policy involvement.	None	Per capita consumption data for beer, wine, and spirits. Prospects for rising rates of problems. Limitations of data sources.
Babor (2015)	Marketing, product design, pricing, with particular detail on marketing strategies.	Product innovations used to negotiate tax incentives. Promotion of marketing selfregulation and other forms of lobbying promoting ineffective policy measures. Legal actions & donations.	SAPROs operating & their activities, including the promotion of partnerships as alternatives to regulation in lobbying and brand building. Other actions by companies.	Analyses impacts on alcohol availability of industry activities in economic, political, CSR spheres.
Esser (2015)	Market research data. Investments. Marketing, sales, and product innovations.	Corrupt payments in India and elsewhere. Alcohol policy contexts in India’s 29 states.	CSR strategy & actions in India & internationally including women’s empowerment programs. Absence of evidence supporting CSR activities.	Trends in alcohol consumption 1990–2010.

*Notes:* CSR = corporate social responsibility; SAPRO = social aspects and public relations organizations.

### Findings on alcohol industry strategies

Although the studies have not generally addressed industry strategy directly as a core object of study, there are nonetheless data available for synthesis within and across studies. There appears to be a high degree of strategic collaboration between companies, as reflected for example in investments in social aspects and public relations organizations (SAPROs) (Babor et al., 2015). The structure of the industry in a particular country or region is highly relevant to patterns of collaboration, although the dynamics of strategic collaborations do not appear simply reducible to economic factors (Jernigan & Babor, 2015). There are patterns of competition and collaboration co-existing, and the balance between the two changes over time and varies in different dimensions of corporate strategy (Babor et al., 2015; Jernigan & Babor, 2015). These African studies identify increased market competition coinciding with greater alcohol production, promotion, new product development, and pricing innovations, alongside an upsurge in the activities of SAPROs, which act as a medium through which companies collaborate in seeking to influence policy.

There are intimate connections between economic and political factors; for example, success in lobbying for tax advantages has important implications for product development, which are in turn closely associated with sales and consumption. Strategically influencing policy in this manner is thus a form of market building (Stockwell & Crosbie, 2001). Further, greater concentration has enabled alcohol industry actors to position their contributions to the local economy as providing rationales for increased involvement within policy-making, potentially facilitating further political influence (Jernigan & Babor, 2015).

The attention given here to interconnectedness of the elements of corporate strategy in the alcohol industry should not, however, diminish the key and perhaps unsurprising major finding that political interests are driven largely by business interests, whether at the company, sectoral, or industry level. These different levels imply potential for conflicts between companies or sectors, and these have been observed in respect to policy positions for example, although such differences appear to be the exception rather than the rule (Giesbrecht, 2006). They also appear to be well managed; the basis for shared interests has been suggested to lie in the oligopolistic structure of beer and spirits industries in particular (Giesbrecht, 2000), and having fewer major companies may facilitate long-term collaborations (Jernigan & Babor, 2015). Recurring key policy issues concern taxation, the regulation of marketing, and product design, and although there is some inconsistency across policy issues, widespread success in policy influence is evident (Giesbrecht, 2006).

CSR is implicated in political strategy when SAPROs promote policies preferred by industry actors when they are lobbying (Babor et al., 2015). CSR also contributes to market building efforts for Diageo in India (Esser & Jernigan, 2015). Such evidence indicates that there is a strong case for questioning the face validity of the claims made about CSR by industry actors. This is especially true because the activities themselves conflict with the evidence on how best to attain the stated goals of reducing alcohol-related harms, with approaches known to be ineffective predominating (Babor et al., 2015; Esser & Jernigan, 2015). Although the data from these studies point toward numerous synergies in strategy development, it is challenging to appreciate the precise contributions made by CSR to business and political strategies with the data in the included studies. The importance of investments in SAPROs to industry-wide political strategy is clearly identified, although there is limited data available at the individual company level on CSR and other elements of strategy.

### Public health implications

The final set of review-level findings to be presented deal with how the included studies analyze the public health implications of industry actor strategy. The growth of alcohol sales has adverse effects on population health by virtue of the evidence that high levels of consumption will produce higher levels of a wide array of alcohol-related harms (Babor, 2010). Babor and colleagues (2015) identify availabilityas a key link in the causal chain postulated in the corporation-induced disease theory, and consider the evidence for Africa as suggestive but not proven in their study. Similarly, Giesbrecht (2006) identifies increased access to alcohol over time as key to increasing overall consumption and heavy drinking. This is not the only pathway, however, and successful influence of policy by industry actors may extend availability or lead to increased harm in other ways (Giesbrecht, 2000). It is also the case that public policy interventions may alter the conduct of industry actors in ways that can benefit population health, for example through tax changes reducing the relative price of lower-alcohol products compared with those containing higher doses (Stockwell & Crosbie, 2001). There are no indications that voluntary measures by industry actors may ameliorate the public health burden (i.e., without the stimulus provided by taxation or other policy measures). According to Jernigan and Babor (2015), “future research should explore their activities [the industry] in greater depth . . . in order to further public health understanding of industry tactics and how best to counter them when they threaten to harm both public health and the ability of governments to adopt effective and evidence-based alcohol policies.” Research could explore how economically and politically motivated actions by industry actors add to the public health burden and how the burden can be reduced by gaining and promoting better understanding of industry strategies and evaluating the effectiveness of efforts to counter the resulting initiatives.

## Discussion

This systematic review included few studies, demonstrating that there is little in the way of a tradition of PHS studies that focus on the integration of business and political strategies in investigating alcohol industry actors. This finding also makes necessary careful attention to the limitations of this study, and particularly how the included studies may be situated in the larger literature. Less stringent inclusion criteria would have allowed a greater number of studies to have been included; not requiring studies to report conceptualization and data on economic and political strategies and health implications, for example, would have facilitated a broader perspective on the nature of the research data available on alcohol industry actors more generally. That was not the aim of the present study. The focus of the present study on the state of current knowledge on integration of the elements of corporate strategy is justified, given the wider business literature and the lack of a prior review of this nature of alcohol industry actors. The low number of studies included, and thus the early stage in the development of this literature, is therefore a major, although unsurprising, finding of this review. The value of this study lies in giving detailed attention to this early research as a platform for developing thinking about how PHS studies in this area may be taken forward. The three older studies may be regarded as foundational to the more recent PHS studies, and it may well be that the 2015 studies come to be viewed as making seminal contributions to the literature. Their value lies in the “big picture” perspective they afford, and they are strong in historical contextualization, making them particularly useful for informing consideration of conflicts of interest between corporate strategies and public health considerations, and how they may best be managed.

### Review-level findings in the context of study objectives and the wider literature

We will now give attention to Study Objectives 1–4 before considering the nature of the need for future research (Objective 5) in the succeeding section. There are clear limitations to the methods reported in these PHS studies, and disparate data sources have been used. Notwithstanding these observations, there are important data available in these studies for understanding alcohol industry behavior, and it is to be anticipated that they will prove very valuable in informing public health responses globally. Although there are few studies, the pathways to adverse impacts on population health, particularly for LMICs, are clear.

One way to address the methodological limitations of the PHS investigations of alcohol industry actors is to consider their coherence with existing evidence and the ways in which they may extend it. The limitations of this review are partly described by the studies excluded. Some had no political data (Ferreira-Borges et al., 2017; Jernigan, 2009; Romanus, 2000), no economic data (Casswell, 2013; Hope, 2006), or no health data (Alavaikko & Österberg, 2000; Dumbili, 2014; Zeigler, 2009). There are also social science–informed studies with more precise objects of investigation (Hawkins et al., 2018; Miller & Harkins, 2010) and historical (Fahey, 1980) and business (Bower & Cox, 2012) studies. The findings examined here are, at a high level of generality, consistent with primary studies included in systematic reviews of CSR (Mialon & McCambridge, 2018), policy involvement (McCambridge et al., 2018), and the review-level syntheses. The existing PHS studies add modestly to the evidence that CSR is integral to political strategies and can be linked to marketing strategies, and that alcohol company political strategies are coordinated with other companies. There is limited data available on how the market development strategies at the individual company level may be configured so as to shape and respond to policy and regulation measures, and to use CSR in so doing.

### Implications for further research

The absence of formal risk of bias assessment constitutes a limitation of this review, and a critical appraisal using conventional instruments might be expected to indicate a high risk of bias among included studies. It is unclear, however, how biases should be conceptualized and assessed in PHS studies. It could be useful to develop guidance to clarify issues concerning study design and risk of bias for future PHS studies of corporate actors. For example, guidance might first identify the circumstances in which PHS studies of industry actors are appropriate to undertake (as opposed to studies using other identified study designs). Guidance could then cover key elements of study design and reporting (Stroup et al., 2017), as appropriate for PHS studies of industry actors. This will, in turn, strengthen the methods used in future PHS studies (Velasco et al., 2014) and permit assessment of the contribution made to the wider research literature. Although informed by the theory of corporation-induced diseases, PHS studies are not designed to test theories. It is suggested here, on the basis of the studies reviewed, that PHS studies may be most usefully regarded in research terms as hypothesis generating and calling attention to issues in need of more in-depth studies using alternative study designs.

There is a considerable body of material for research agenda setting in PHS studies of alcohol industry strategies. For example, the means of forging and maintaining successful cooperation among industry actors, particularly in oligopolistic markets, warrants careful study. Case study designs may be most appropriate for such studies (Yin, 2012). The effects of alcohol marketing on public support for alcohol policy measures are largely unstudied and could be investigated with survey designs. Better understanding of the synergies and intimacy of the connections between economic and political interests will permit new insights into industry strategies, as will data on the public health impacts associated with successful attempts to influence policy by industry actors. CSR also needs to be further studied to develop understanding of company level and industry-wide strategy formation, and SAPROs appear particularly important in this regard. The growing concentration among global producers calls for analytic approaches rooted in political economy (Hawkins et al., 2018), management studies, and other disciplines that are well placed to appreciate the connections between the economic, political, and CSR elements of integrated strategies within and across industries dominated by a small number of large transnational corporations.

Research advances in PHS studies and in other study designs can be translated into new understanding of how to advance public health goals, and studies that rigorously synthesize data from multiple studies will be particularly important in so doing. The rigor of new primary research studies will be aided by using recognized study designs, such as natural experiments or case studies, and the methodological guidance for the conduct of such studies. The lack of transparency around alcohol industry activities targeted at influencing policy decisions, and a lack of transparency around internal company decision-making and policy formation itself, pose challenges to research (Giesbrecht, 2006). Access to insider information (Giesbrecht, 2006) may therefore be helpful. As well as studies undertaken within particular disciplines or with specific foci, multidisciplinary data may be synthesized in future reviews of PHS implications, although these should be expected to be complex endeavors. They are needed, however, as the combination of empirical and conceptual data from PHS studies with those originating from social sciences offers one way to link rigorous scrutiny of alcohol industry actors to investigations of other corporate sectors. The similarities in strategy formation across corporate sectors may far outweigh the differences, meaning that there is much to learn in so doing. This is perhaps particularly true of the tobacco industry, given the similarities in the strategic problems faced and the structures of the industries. Comparative analyses of such industries as food and gambling could also be very valuable.

PHS studies may be most useful for public health actors when they are conducted close in time to the actions being studied, and where these are self-evidently important to global efforts to reverse NCD trends in LMICs and elsewhere. The 2015 studies of Africa and India (Babor et al., 2015; Esser & Jernigan, 2015; Jernigan & Babor, 2015) provide key data to inform decision-making by policy actors about how to manage alcohol industry actors’ attempts to influence policy. These types of studies require extensive monitoring for data collection, and partnerships between researchers and nongovernmental organizations and other actors who are well placed for such monitoring may be needed to support the further development of PHS studies of industry actors.

### Conclusion

Producing, selling, and marketing harmful products requires careful and effective high-level political operations, and alcohol industry actors have been active in developing partnerships with governments across the world. In some ways, studies of alcohol industry actors are like “shooting fish in a barrel”; because the scientific literature is so underdeveloped, there are many important contributions to be made. We need more PHS studies of industry actors—many more. Alcohol industry strategies appear highly integrated within and across companies and other organizations, and better understanding of precisely why and how this is so, and where it is not, should be a key task for further study. It is concluded that PHS studies have a valuable role in identifying aspects of industry conduct that warrant more detailed and carefully designed studies, and in elucidating the global health implications and thereby assisting efforts to reduce the global burden of disease caused by alcohol.

## Acknowledgment

The authors are grateful to Melissa Mialon for various contributions to the early conduct of this study, including the first wave of data collection.
